# Research Progress of Ferroptosis Regulatory Network and Bone Remodeling in Osteoporosis

**DOI:** 10.3389/fpubh.2022.910675

**Published:** 2022-06-30

**Authors:** Chunlu Yan, Jinlong Zhang, Fangyu An, Jiayu Wang, Yao Shi, Lingqing Yuan, Donghui Lv, Yanzhen Zhao, Yongfeng Wang

**Affiliations:** ^1^School of Traditional Chinese and Western Medicine, Gansu University of Chinese Medicine, Lanzhou, China; ^2^The First Clinical Medical College, Gansu University of Chinese Medicine, Lanzhou, China; ^3^Teaching Experiment Training Center, Gansu University of Chinese Medicine, Lanzhou, China; ^4^School of Basic Medicine, Gansu University of Chinese Medicine, Lanzhou, China

**Keywords:** ferroptosis, osteoporosis, bone formation, bone resorption, network regulation

## Abstract

Ferroptosis was induced the programmed cell death with iron overload Fenton reaction. Currently, ferroptosis has not been studied thoroughly. Existing studies have confirmed that ferroptosis involves the metabolisms of the Fe, lipids, amino acid, each mechanism is mutually independent but interrelated, and they are formed a complex regulatory network. Other evidence supports that ferroptosis is participated osteoporotic bone remodeling, predominantly affecting the interaction between bone formation and bone resorption, explicitly bone resorption exceeded bone formation. Based on previous studies, this review will summarize the regulatory network mechanism of ferroptosis on bone remodeling and reveal the role of ferroptosis in osteoporosis (OP).

## Introduction

Osteoporosis (OP) is a metabolic disorder, which is characterized by the decrease of bone mass, and the increase of bone brittleness (1). With the aggravation of the nation's aging population, the incidence of OP is increasing. By 2020, epidemiological methods were used to analyze the prevalent OP ratio by the Chinese Center for Disease Control and Prevention (CDC), the data indicates that the incidence of people above 50 was 19.2% (2), and it is expected that the number of OP patients suffering from brittle fractures in developing country will reach 6.26 million by 2050 (3), OP has become an important issue in public health in China. Bone is an active metabolism tissue, which constantly undergoes bone remodeling. The mutual restriction between bone formation and bone resorption is a critical factor to ensure bones healthy and integrity during the life cycle. Therefore, the imbalance of its might be the main reason for the occurrence and development of OP (4).

The imbalance of bone formation and resorption is considered the main mechanism of osteoporosis. Bone formation is involved with a large number of osteoblasts (OB) under the periosteum. OB release bone matrix rich in collagen type I and participate in the hydroxyapatite crystal formation in collagen fiber deposition process, namely bone mineralization process, eventually forming new bone. Bone resorption occurs mainly on the bone surface, by the osteoclasts (OC) on bone metabolism in continuous absorption. OB mediates bone formation through runt-related transcription factor 2(Runx2). Runx2 is a critical regulatory factor promoting OB maturation in early differentiation. Further, it regulates osteoblastic extracellular matrix such as osteopontin (OPN), osteocalcin (OCN), bone sialoprotein (BSP) in late differentiation. The expression and transcription of alkaline phosphatase (ALP) and BSP promote the maturation and mineralization of OB (5). Further, there are signaling pathways that participate in regulation, such as Wnt channel, mitogen-activated protein kinase (MAPK) channel, bone morphogenetic protein (BMPs) channel, peroxisome proliferator activated-receptors (PPARs) channel, and Notch channel. These form a complex network structure and precisely regulate bone formation (6).

Osteoclast, derived from macrophage-mononuclear lineage, is a special myeloid cell. Its differentiation and function are mainly regulated by macrophage colony-stimulating factor (M- CSF), receptor activator of nuclear factor kappa B(RANK) (7). M-CSF binds to the M-CSF receptor, which is promote the proliferation and differentiation of OC and the continuous expression of RANK. RANK binds to transmembrane protein nuclear factor-κB receptor activating factor ligand (RANKL) to activate RANK/RANKL, TRAF6/RANKL/MAPKs, TRAF6/ RANKL/NF-κB, Wnt/RANKL/RANKL, and JAK2/STAT3/RANKL. Multiple signaling pathways stimulate the differentiation and proliferation of OC. OC destroys the mineral and collagen matrix by secreting matrix metalloproteinases (MMPs), cathepsin K (CtsK), H^+^ and Cl^−^, and eventually forming bone lacunae. OC plays the role of bone-eating. However, osteoprotegerin (OPG), a natural antagonist of the RANKL-RANK pathway, is a glycoprotein that can inhibit OC differentiation. OPG can inhibit OC differentiation by competing with RANKL and binding to RANK. Actin binding protein L-plastin (LPL) can regulate the osteophagy function of osteoclasts. Trap, osteoblasts, and bone mass increased after Li knocked out LPL. LPL deletion increased the number of preosteoclasts, and preosteoclasts released platelet-derived growth factor-BB to promote bone formation. In addition, LPL inhibitor oroxylin A can improve bone loss caused by ovariectomy and accelerate fracture healing in mice through PI3K/AKT signaling pathway (8). Recent studies have confirmed that bone marrow adipocytes (BMAs) derived from bone marrow mesenchymal stromal cells are also the key regulatory factors of osteoclasts. Excessive BMAs promote osteoclast differentiation and play a role in phagocytosis. It is worth noting that BMAs can also express RANKL, suggesting that BMAs may participate in bone remodeling through RANK/RANKL signaling pathway (9). Other studies have reported that adipose cells are an important way to mediate osteoclasts to destroy trabecular bone and absorb cancellous bone during remodeling through RANK/RANKL. Targeted regulation of bone marrow fat is an effective method to prevent pathological bone loss (10). It has been reported that BMSCs derived from bone marrow have immunomodulatory effects in tissue microenvironment, BMSCs exosomes derived from bone marrow can significantly reduce the release of IL-10 and TGF-β from peripheral blood mononuclear cells of asthma patients than BMSCs themselves, thus alleviating the symptoms of asthma attack. Whether BMSCs exosomes derived from bone marrow have similar effects in regulating bone remodeling needs further study and confirmation (11). Bone remodeling is maintained in equilibrium by mutual restriction of OB and OC (12).

Ferroptosis (FPT) is a form of cell death, which is characterized by the increase of the mitochondrial membrane density and the shrinkage of the mitochondrial. The morphological characteristics of ferroptosis shows that mitochondria volume decreases, mitochondrial membrane density increases, mitochondrial outer membrane integrity is destroyed, mitochondrial cristae dissolve and disappear (13). It is intimately connected to the development and progression of tumor, non-alcoholic fatty liver, parkinson's syndrome, congestive heart failure, etc. (14). In 2012, Dixon et al. (15) reveled that the oncogenic RAS-selective lethal small molecule erastin triggers a unique iron-dependent form of non-apoptotic cell death, which is dependent upon intracellular iron, but not other metals, and is morphologically, biochemically, and genetically distinct from apoptosis, necrosis, and autophagy, this new cell death pattern was named ferroptosis. In 2021, Tang reveled that ferroptosis was caused by iron-overload, glutathione peroxidase 4(GPX4)/glutathione (GSH), and lipid peroxidation (16).

Recently, the regulatory effect of ferroptosis on osteoporosis is becoming a focus of research. Current studies have confirmed that the following biochemical metabolisms are involved in osteoporosis during ferroptosis: (1) The number or function of iron transporter is abnormal, and the dynamic equilibrium state of the iron level in the human body is broken. A massive accumulation of Fe produces lots of ROS by means of the fenton reaction, resulting in lipid peroxidation and damaging the cell biofilm system. Further, it affects the differentiation and proliferation of OB and OC by regulating the signaling pathway of bone metabolism; (2) The decrease of cysteine intake in GSH synthesis and the depletion of GSH in cells lead to the decrease of GPX4 activity. Therefore, it cannot catalyze the reduction reaction of lipid peroxidation products in time, damaging the integrity of cell membrane, osteogenic ability, and bone microstructure of OB; (3) FTH and NCOA4 can act together to cause autophagy degradation of ferritin and release a large amount of Fe^3+^ to initiate ferroptosis. These biochemical metabolisms, osteoporotic bone formation, and bone resorption-related signal pathways are interrelated and mutually regulating, affecting the occurrence and development of osteoporosis. The ferroptosis regulatory network regulates the bone remodeling cycle of osteoporosis, leading to dynamic unbalance of bone formation, bone resorption. This leads to less bone formation than bone resorption, thus promoting bone loss (17). In view of this, this review will summarize the regulatory network mechanism of ferroptosis on bone remodeling and provide theoretical basis for the prevention and treatment of osteoporosis.

### GPX4/GSH Levels Change of Ferroptosis

Glutathione peroxidase 4 (GPX4) is a central regulator, which is the body's lipid antioxidant system. Glutathione (GSH) is used as a cofactor to convert peroxide (R-OOH) into alcohol (R-OH) and reduce the toxicity of lipid peroxides to protect biofilm systems from ferroptosis damage. However, GSH depletion in the body affects the activity of GPX4, which is a necessary condition for the occurrence of ferroptosis. GSH is a key antioxidant in oxidative stress response and is synthesized by L-cysteine, glycine, and glutamate, among which the concentration of the least amount of cysteine can limit the synthesis rate of GSH (18). GSH originates from multiple synthetic pathways, including Glutathione synthetase (GSS) and nicotinamide adenine dinucleotide phosphate (GSS). In the presence of NADPH, Glutathiol (GSSG) was reduced to GSH, supplemented with a small amount of GSH. Secondly, the trans-vulcanization pathway takes the sulfur donor methionine as the substrate and catalyzes cystathionine-γ-lyase (CGL) and cystathionine-β-synthase (CBS) to produce cysteine, which participates in the synthesis of GSH. It plays an antioxidant role (19). Thirdly, XC-system is a cystine-glutamate reverse transporter protein formed by disulfide bond linking heavy chain SLC3A2 and light chain SLC7A11. It mediates the exchange of cystine and glutamate in and outside the cell in a ratio of 1:1. Extracellular glutamate level will affect the transport rate of the XC-system, and a high concentration of glutamate will inhibit cystine uptake and GSH synthesis, thus affecting the activity of GPX4 and leading to ferroptosis (20). Erastin, a compound that induces ferroptosis, inhibits the XC-system in a regulatory manner similar to SAS. There is evidence that p53 inhibits the XC-system by down-regulating SLC7A11. It will reduce cysteine uptake. Therefore, structural and functional impairment of the XC-system is a key factor for ferroptosis (21).

### GPX4/GSH and Bone Formation

Depletion of GSH and decreased GPX4 activity can lead to ferroptosis and induce osteoporosis. Ma et al. (22) reported that large amounts of ferroptosis lipid peroxides accumulate in osteoblasts of osteoporotic patients with type 2 diabetes. These lipid peroxides are linked to the down-regulated expression of GPX4 and SLC7A11 in OB mitochondria and XC-system. However, when Mc3t3-e1 cells treated with diverse melatonin densities, the above phenomenon was reversed to varying degrees. By activating the NRF-2/HO-1 antioxidant channel, melatonin reduces ROS levels, upregulates SLC7A11 levels, and increases GPX4 activity. Further, it alleviates the toxicity of lipid peroxides to protect the biofilm system from ferroptosis, improving the osteogenic ability and bone microstructure of OB. Long-term use of glucocorticoids can cause the decrease of bone mineral density, leading to secondary osteoporosis. Studies have found that glucocorticoids can induce OB iron overload and inhibit antioxidant capacity. Yang et al. used EC-EXos, an exosome secreted by endothelial cells, to intervene in the process of OB ferroptosis induced by glucocorticoids. GPX4 and FTH were up-regulated, ASCL4 expression was down-regulated, and the effect was similar to that of ferroptosis inhibitors DFO and FER-1. Further studies found that glucocorticoid-induced autophagy-related proteins such as NCOA4, LC3, P62, and Beclin-1 were also up-regulated. FTH interacts with ncoa4, which was resulted in autophagic degradation of ferritin and release a large amount of Fe^3+^ to cause ferroptosis of OB. Ferroptosis is also inhibited when the autophagy inhibitors 3-MA and RPA are used. Therefore, EC-EXos can reduce the degree of damage caused by ferroptosis by inhibiting iron overload in the phagocytic dependence of ferritin (23). In another study on the mechanism of lung cancer, it was found that lncRNA H19 may inhibit the transcription level of FTH1 by competitively binding miRNA-19b-3p, so as to protect lung cancer cells from iron death injury, thus weakening the effect of anticancer drugs (24). It can be seen that iron death has potential physiological function and pathological mechanism in the process of tumor suppression and immune monitoring, and has a clear guiding role for targeted therapy (25). The strategies to promote bone formation was regulated iron ion level by regulating iron metabolism related signal pathway to prevent ferroptosis, its strategies was also increased the activity of GPx4 to remove lipid peroxide, it will probably be the key basis for future drug development in osteoporosis.

### Iron Transport-Related Proteins and Bone Formation

Hepcidin (HEP) is a regulating hormone in iron homeostasis, synthesized and released by liver cells. It can bind with the transmembrane protein FPN receptor to inhibit the entry of cellular iron into circulation (26). So far, FPN is the only iron output protein in vertebrates. If the activation of HEP-induced FPN is insufficient or ineffective, iron overload or iron deposition in bones may occur in the body, producing lots of ROS, and ultimately leading to osteoporosis (27). Jiang et al. observed bone loss, bone microstructure damage, bone mineralized area reduction with various density of ferric ammonium citrate (FAC) in OB of zebrafish embryos (28). Further, the results showed that the Runx2, an essential regulator of osteogenic differentiation, was increased, ROS-induced lipid peroxidation was decreased, and bone mineralization area improved significantly with the increase of HEP concentrationg (28). These results suggest that HEP overexpression can prevent iron overload and inhibit bone formation.

It has also been reported that BMP/SMAD signaling pathway can regulate the expression level of HEP (29). Further, the increased iron concentration stimulates hepatic endothelial cells to produce BMP2 and BMP6 in a paracrine manner. BMP2 and BMP6 regulate the expression of HEP through downstream SMAD protein, but the mechanism has not been clarified, and further studies are needed. Rauner (30) studied the HEP downstream target transferrin receptor 2 (Tfr2) mechanism in bone metabolism using a mouse model with Tfr2 knockout bone marrow transplantation. The results demonstrated that Tfr2 specifically activated BMP through BMP/p38MAPK signaling pathway and increased the expression of recombinant sclerostin (SOST), a natural inhibitor of the Wnt signaling pathway. This suggests that Tfr2 may transport a large amount of iron into osteoblasts, and iron overload leads to more ROS in the Fenton reaction. Activation of the BMP/p38MAPK channel effectively inhibits OB activity, and increases the expression of SOST. It competitively binds low-density lipoprotein receptor-associated protein 5/6 (LRP5/6) and prevents Wnt signaling pathway transduction, thus inhibiting the differentiation and maturation process of OB. Other studies have found the expression of iron transporter proteins FLT, FPN1, and TFR1 genes in mouse bone tissue after IRP2 deletion. This is a sparse disorder of bone trabecular bone, leading to the reduced iron content and decreased expression of bone formation markers, which are alkaline phosphatase (ALP), osteocalcin (BGP), collagen I α (Colα1). However, the expression of tartrate-resistant acid phosphatase (TRAP), and Ctsk are increased (31). Therefore, IRP2 deficiency may inhibit the transport of iron transporter and leads to iron deficiency, affecting bone metabolism. However, the mechanism is unclear, and more detailed studies are needed to explain this finding in the future. Similarly, Yao observed that the expression level of type II collagen was also decreased in iron death model of arthritis (OA) mice induced by IL-1 and FAC, which was consistent with previous research results. The increased expression of matrix metalloproteinase-13 (MMP13) promoted cartilage degradation and aggravated cartilage degeneration. In addition, Nrf-2/HO-1 antioxidant system was inhibited during iron death. When Nrf-2 was knocked out, the activity and expression level of GPX4 protein decreased, which aggravated the degree of iron death. These results suggest that Nrf-2/HO-1 antioxidant system and iron death can be mutually regulated under inflammatory conditions (32). These findings suggest that Fe overload can result in bone remodeling disorder, as bone resorption is greater than bone formation. Therefore, the iron metabolism balance plays a vital role in osteoporosis.

### Ferroptosis Related Signaling Pathways and Bone Formation

Iron metabolism is regulated by many signaling pathways during ferroptosis. Ma (22) found that activation of nuclear factor E2-related factor 2(NRF2)/Heme Oxygenase-1(HO-1) channel significantly reduces the ferritin level and alleviates oxidative stress. Further, this inhibits ferroptosis, thereby increasing bone formation. Under physiological conditions, NRF2 binds to the negative regulatory factor Kelch-like ECH-associated protein 1(KEAP1) to form the NRF2- KEAP1 complex, reducing the activity of NRF2 in the cell. Iron overload produces a large number of ROS. Under stress conditions, the NRF2-KEAP1 complex degrades, and NRF2 enters the nucleus after activation. NRF2 binds with MAF protein and acts on the antioxidant reaction elements. NRF2 activates the downstream glutathione peroxidase (GSH-PX) and superoxide dismutase (SOD), which is initiated the cellular peroxidation and defense mechanism. Further, it removes harmful substances such as ROS, reducing the damage to OB.

Other studies have found that phosphoinositide3-kinase (PI3K)/Serine threonine kinase (AKT) shannel also participated in the differentiation and maturation of OB (31). Xia (33) revealed that iron overload significantly inhibited OB proliferationm, induced apoptosis. Reduced the expression of specific phosphatase 14(DUSP14), and DUSP14 played a protective role in bone metabolism mediated by Forkhead box O3(FOXO3a). p-FOXO3a is regulated by phosphorylated protein kinase B(p-Akt). The results showed that the PI3K/AKT/FOXO3a/DUSP14 channel may be the protection mechanism of oxidative stress in osteoporosis.

Tian (34) found that iron-overload-induced ROS formed a positive feedback loop through RIPK1/RIPK3/MLKL signaling pathway to promote OB necros. Further, the phosphorylation of RIPK1 and RIPK3 during ROS accumulation is increased, and then the two are combined to form a necrotic complex. Phosphorylated MLKL is translocated to the plasma membrane to perform necrotizing apoptosis. In conclusion, iron uptake, storage, excretion disorders, and abnormal expression of iron transport-related proteins IRP2, FLT, FPN1, TFR1, TFR2, and HEP can lead to changes in iron concentration. Through the Fenton reaction, iron overload can produce a large number of ROS, which is the key target of bone remodeling imbalance. The key targets are mainly regulated by many signaling pathways, including BMP/p38MAPK, BMP/SMAD, NRF2/HO-1, PI3K/AKT/FOXO3a/DUSP14, RIPK1/RIPK3/ MLKL, and Wnt.

### Ferroptosis and Bone Resorption

Many case reports and basic studies have shown that ferroptosis can promote OC differentiation and bone resorption, ferroptosis induced by iron overload. It causes severe damage to bone microstructure, but an iron-chelating agent can inhibit OC differentiation and increase OB activity. Bone cells differentiated from mesenchymal stem cells and OB are embedded in the mineralized matrix. They coordinate the functions of OC and OB by secreting OC-promoting factor RANKL and anti-OC factor OPG, indirectly regulating bone remodeling. It has been reported that a high iron level can induce osteocyte apoptosis, significantly promote the RANKL production, increase the ratio of RANKL/OPG in osteocytes, they ultimate enhancing the differentiation and bone-eating function of OC (35). In another study, after iron overload, there were obvious bone abnormalities, the calcium content and bone mineral density of bone tissue decreased significantly, and the bone microstructure was damaged in osteoclast (36). Further, the expressions of OPG, osteocalcin, and serum antioxidant enzyme SOD were reduced. However, the expressions of ROS, MDA, RANKL, and IL-6 were all raised in osteoclast (36), suggesting that ROS produced by iron overload Fenton reaction may promote the expression of RANKL and enhance bone resorption.

The other evidence reveal that RANKL is downstream of IL-6 and regulates the differentiation and bone resorption of OC. Yet, the regulatory role of IL-6 in ferroptosis-caused bone damage needs to be studied further in detail. Under physiological conditions, iron forms Fe-S clusters in mitochondria, which participated in mitochondrial biogenesis and electron transfer. When erastin, a ferroptosis inducer, is applied to cells, the negative ion channel protein VDAC2/3 is opened, resulting in a large influx of iron to produce ROS. ROS is required for RANKL, which is stimulated the mononuclear macrophage lineage differentiated into OC. Paul (37) reported that the iron-chelating agent DFO could reduce iron content in cells. It significantly inhibits OC differentiation by inhibiting the electron transport chain and MAPK signaling pathway. MAPK pathway is one of the main participants in OC differentiation. DFO may affect the expression levels of downstream NFATc1, C-FOS, and c-Myc, which was regulated the proliferation and differentiation of OC. Therefore, RANKL is the key target of ferroptosis in regulating bone resorption, which provides a theoretical basis for developing new drugs for osteoporosis. In future studies, whether lipid metabolism and amino acid metabolism can regulate bone resorption will become the main research area to find multi-link, multi-target, comprehensive treatment of osteoporosis drugs (38).

In conclusion, iron overload can inhibit OB activity and differentiation, promote OC differentiation, and eventually cause bone remodeling disorder. It can be seen that iron metabolism balance plays an important role in osteoporosis. The regulation of iron metabolic balance may become a key target for the treatment of osteoporosis in the future.

## Regulation of Metabolism on Ferroptosis

Studies have confirmed that biological metabolic processes regulate the occurrence and development of various diseases during ferroptosis, these biological metabolic processes involve the metabolisms of the Fe, lipids, and amino acid (39).

### Regulation of Abnormal Iron Metabolism on Ferroptosis

Iron is a trace mineral essential, which maintains the body's physiological functions, and it is also involved in cell growth, cell division, DNA repair, and other life metabolic activities. Iron in the human body mainly comes from meat as heme irons (Fe^2+^) and non-heme irons (Fe^3+^) from plants. Fe^2+^ can combine with proteins and participate in various REDOX reactions, while Fe^3+^ is the chief form of body's iron transportation (40). After food digestion, absorbed iron through intestinal epithelial cells (ICE) forms intron under the action of transferrin receptor (TfR1). Fe^3+^ in the endosomes is reduced to Fe^2+^, which regulated the six-transmembrane epithelial antigen of the prostate 3(STEAP3) (41). Then, divalent metal-ion transporter-1 (DMT1) binds to Fe^2+^, and it is transported into the cytoplasm, releasing Fe^2+^ into the unstable iron pool. A portion of Fe^2+^ in the iron pool combines with poly RC binding protein (PCBP) to participate in iron metabolism, storage and transport, while the balance of Fe^2+^ maintained by the other portion of Fe^2+^ binds to reducing cysteine residues of GSH (41).

*In vivo*, excessive Fe^2+^ reacts with ferritin heavy chain 1 (FTH1) to form Fe^3+^, which combines with Ferritin light chain (FTL) to form a complex stored in cells (41). Further, excessive Fe^2+^ can also combine with transferrin (TF) of cell membrane and oxidize to Fe^3+^, forming TF-Fe^3+^ to be released into the extracellular blood transport. These processes keep the intracellular iron in the normal range. Iron regulatory proteins (IRPs) are sensors of iron concentration in the human body. There are two types of human IRPs; IRP1 and IRP2. When iron concentration fluctuates greatly, IRPs regulate the iron transporter expression by binding to the stem-loop structure of ferritin mRNA. The human body maintains a dynamic equilibrium of iron levels through iron input, storage, and outflow (41).

**Figure 1:**
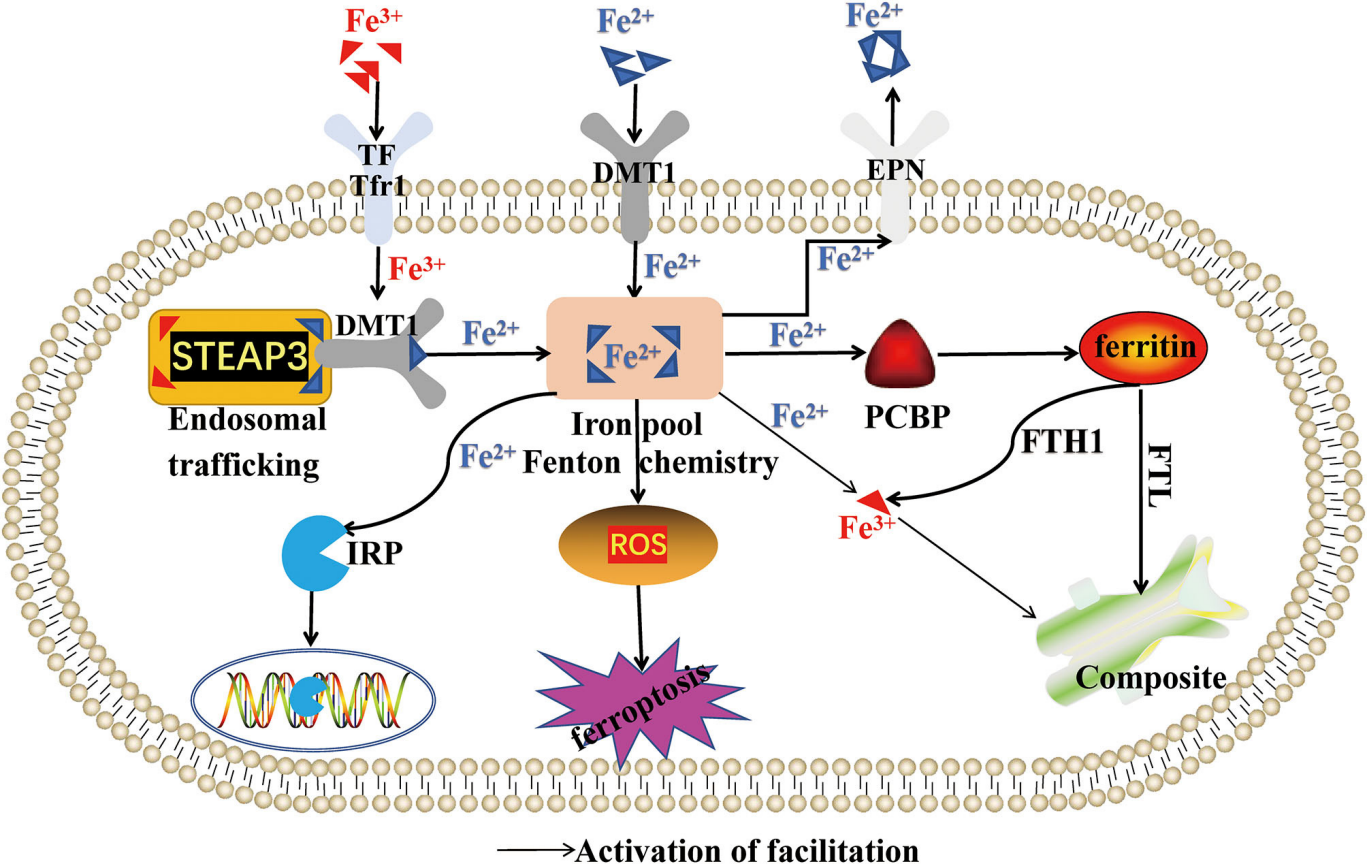
Regulation of abnormal iron metabolism on ferroptosis. This figure summarizes the regulatory effect of Fe^2+^ and Fe^3+^ on ferroptosis. The black arrow represents the activation of the regulatory role of Fe^2+^ and Fe^3+^.

In ferroptosis, iron homeostasis is broken, resulting in a continuous increase of free iron content, excessive activation of oxidative phosphorylation pathway of mitochondria *in vivo*, and the generation of ATP and reactive oxygen species (ROS). Excess iron in the body can also provide electrons for H_2_O_2_ and O_2_ to produce hydroxyl radicals (OH) and superoxide anions (O^2−^), which can degrade lipids, proteins, and nucleic acids in cells, directly damaging the normal structure of cells. Further, iron participates in forming enzymes and subunits in mitochondrial respiratory chain. Excessive iron promotes mitochondrial synthase and subunits, which generate many ROS through Haber-Weiss reaction and Fenton reaction, resulting in ferroptosis (42). However, iron chelating agents deferrone and deferramine (DFO) can remove excess iron and reduce ROS oxidative damage to cells, inhibiting the ferroptosis process. Studies have proved that ferroptosis is often accompanied by ferritinophagy. The interaction of FTH and nuclear receptor coactivator 4 (NCOA4) causes ferritin degradation and releases a large amount of Fe^3+^, leading to ferroptosis. However, ferroptosis can be alleviated by inhibiting autophagy or NCOA4 (43). These results reveal that iron metabolism plays a pivotal regulating role in ferroptosis injury. Therefore, regulation of iron metabolism-related signaling pathways can effectively remove ROS produced by iron metabolism disorder, which may become a key basis for future ferroptosis drug development (Figure 1).

### Regulation of Abnormal Lipid Metabolism on Ferroptosis

Lipid metabolism is associated with ferroptosis. It is generally believed that biofilm components, phosphatidyl ethanolamines (PE) and polyunsaturated fatty acid (PUFA), can induce Lipid peroxidation (LPO). LPO can produce free radicals, malondialdehyde (MDA), 4-hydroxynonenal (4-HNE), and Lipid hydroperoxide (LOOH) to damage the biofilm system. In the biofilm system, PUFA-PE is synthesized from adrenoyl (AdA) and arachidonoyl (AA) containing PUFA. First, adrenoyl (AdA) and arachidonoyl (AA) are synthesized into free fatty acids by acyl-CoA synthetase long-chain family member 4 (ACSL4). Then, free fatty acids (FFA) interact with acetyl coenzyme A (COA-SH) to produce Lipoacyl coenzyme A. Finally, lipoacyl coenzyme A which was esterified by lysophosphatidylcholine acyltransferase 3 (LPCAT3), combines with phosphatidyl ethanolamines (PE) to form PUFA-PE (44). Lipoxygenases (LOXs) are the executor of lipid peroxidation of PUFA-PE. LOXs act on phosphatidylethanolamine binding protein 1 to expose the ferroptosis oxidation site (45), producing ROS and phospholipid hydroperoxides and forming oxygen-free radical chain reactions. As a result, ROS concentration exceeds the physiological limit and damages biofilm structure and physiological function, resulting in ferroptosis pathophysiological changes.

**Figure 2:**
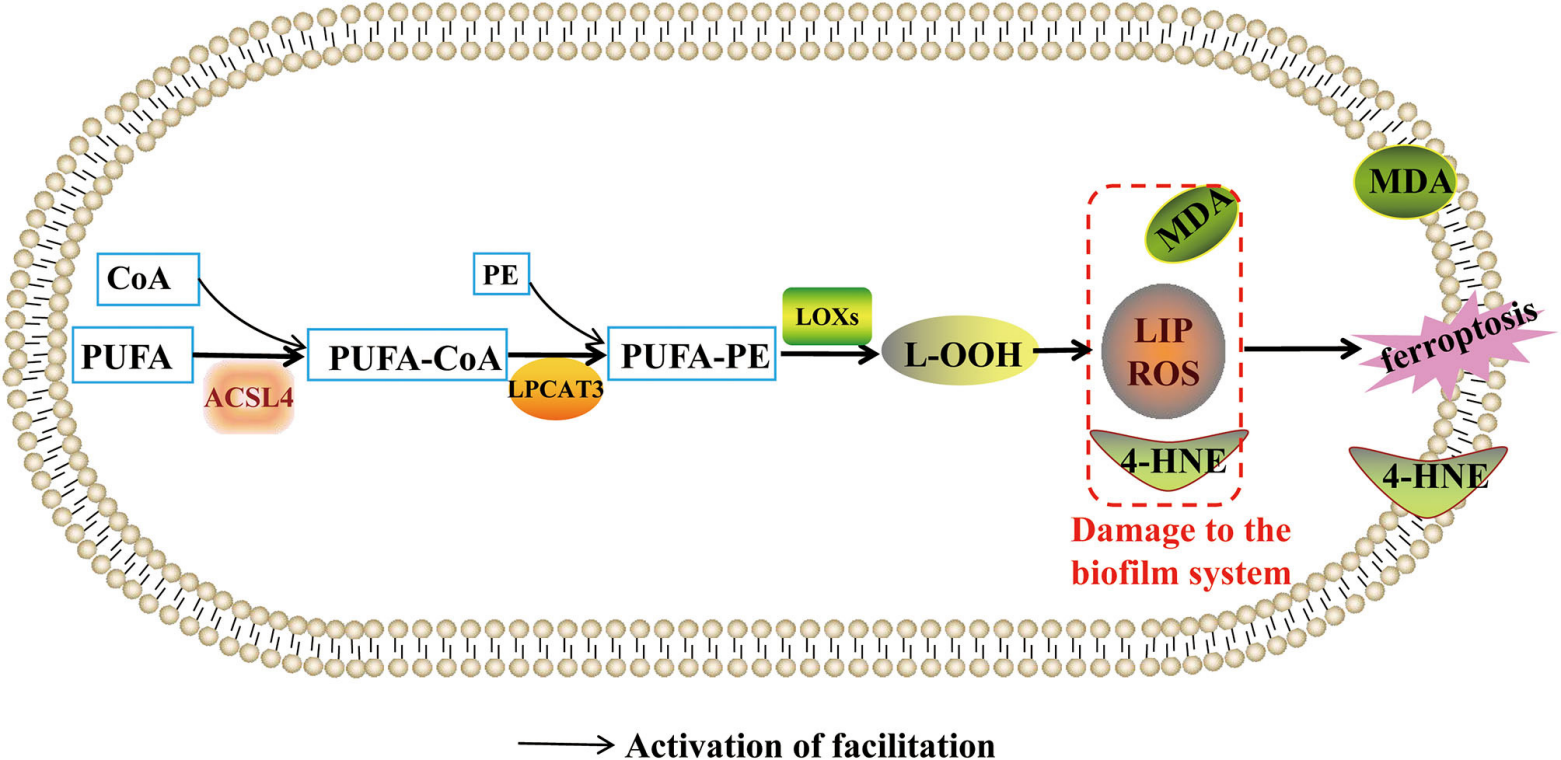
Regulation of abnormal lipid metabolism on ferroptosis. This figure summarizes the regulatory effect of lipid metabolism on ferroptosis. The black arrow represents the activation of the regulatory role of lipid metabolism.

The key regulatory targets of ferroptosis lipid metabolism are ACSL4, LPCAT3, and LOXs. Ma (46) found that ACSL4 is highly expressed in ovarian cancer. Further, up-regulated Mir-424-5P inhibits ACSL4 expression by directly binding to ACSL4 functional region 3'-URT, thereby alleviating ferroptosis induced by RSL3 and Erastin. Therefore, inhibition of ACSL4 expression might be prevent ferroptosis-related diseases. LOXs is a type of ferroprotease whose activity depends on the concentration of Fe^2+^. Some studies found that using the LOXs inhibitor baicalin or silencing the expression of the LOXs gene can reduce the degree of ferroptosis. Therefore, ACSL4, LPCAT3, and LOXs might be the entry point to regulate ferroptosis. Hence, new target molecules regulating the expression of ACSL4, LPCAT3, and LOXs are attempted as the basis for the ferroptosis prevention drugs, providing new ideas to study ferroptosis mechanisms (Figure 2).

### Regulation of Abnormal Metabolism of Amino Acids on Ferroptosis

**Figure 3:**
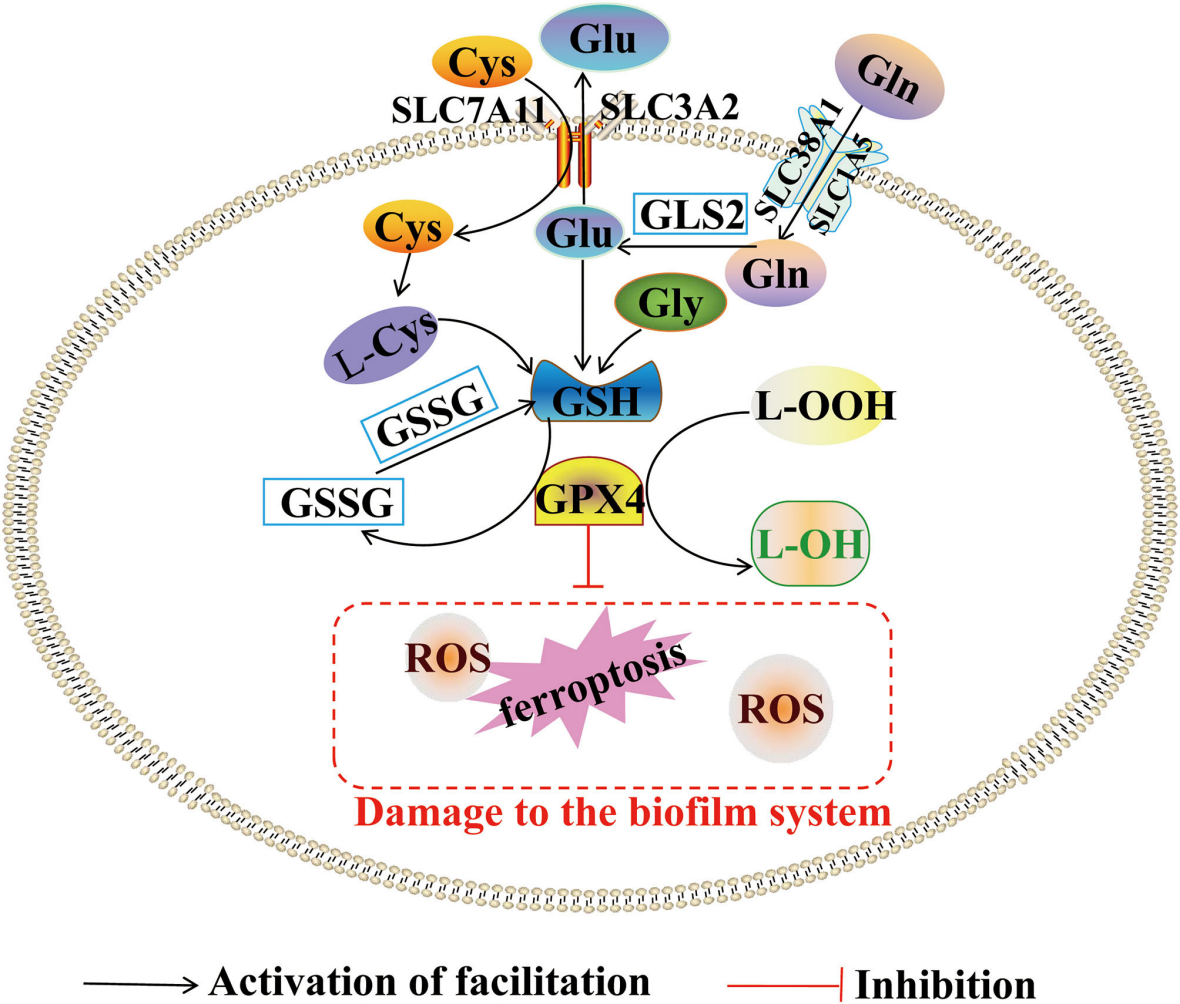
Abnormal metabolism of amino acids and ferroptosis. This figure summarizes the regulatory effect of amino acids metabolism on ferroptosis. The black arrow represents the activating role of lipid metabolism. The red inhibition arrow represents the inhibiting role of lipid metabolism.

The key regulatory targets of iron-dead mediated amino acid metabolism are GPX4 and cysteine. Studies have confirmed that acute kidney injury (AKI) is associated with ferroptosis. According to RNA sequence analysis, activated transcription factor 3 (ATF3) blocks the XC-system, affecting the activity and expression level of GPX4. The high expression of ATF3 promotes the accumulation of many ROS, MDA, and other lipid peroxides, which exacerbates the degree of ferroptosis in proximal renal tubular epithelial cells (47). Recent results showed that ferroptosis involves natural tumor suppression mechanism. Lei (48) reported that ferroptosis regulatory proteins GPX4, SLC7A11, and ACSL4 were increased simultaneously in the experiment of patient-derived xenografts (PDX) mice model treated with ionizing radiation. The occurrence of ferroptosis was inhibited by GPX4 and SLC7A11, while promoted by ACSL4 depending on the regulating PUFA-PE synthesis. This result is paradoxical. Further studies have found that ionizing radiation-induced ACSL4 expression is expressed more than GPX4 and SLC7A11. Therefore, the GPX4 and SLC7A11 may serve as a negative feedback protection to prevent excessive ferroptosis. The discover suggest that GPX4 and cysteine regulate the iron and amino acid metabolism. The findings also suggest that adjusting the expression level of GPX4 and cysteine can effectively prevent and treat tumor diseases, adjusting the expression level of GPX4 and cysteine using iron treatment promotes ferroptosis leading to tumor death. These measures could be the key basis of cancer prevention and control (Figure 3).

## Conclusion and Outlooks

**Figure 4:**
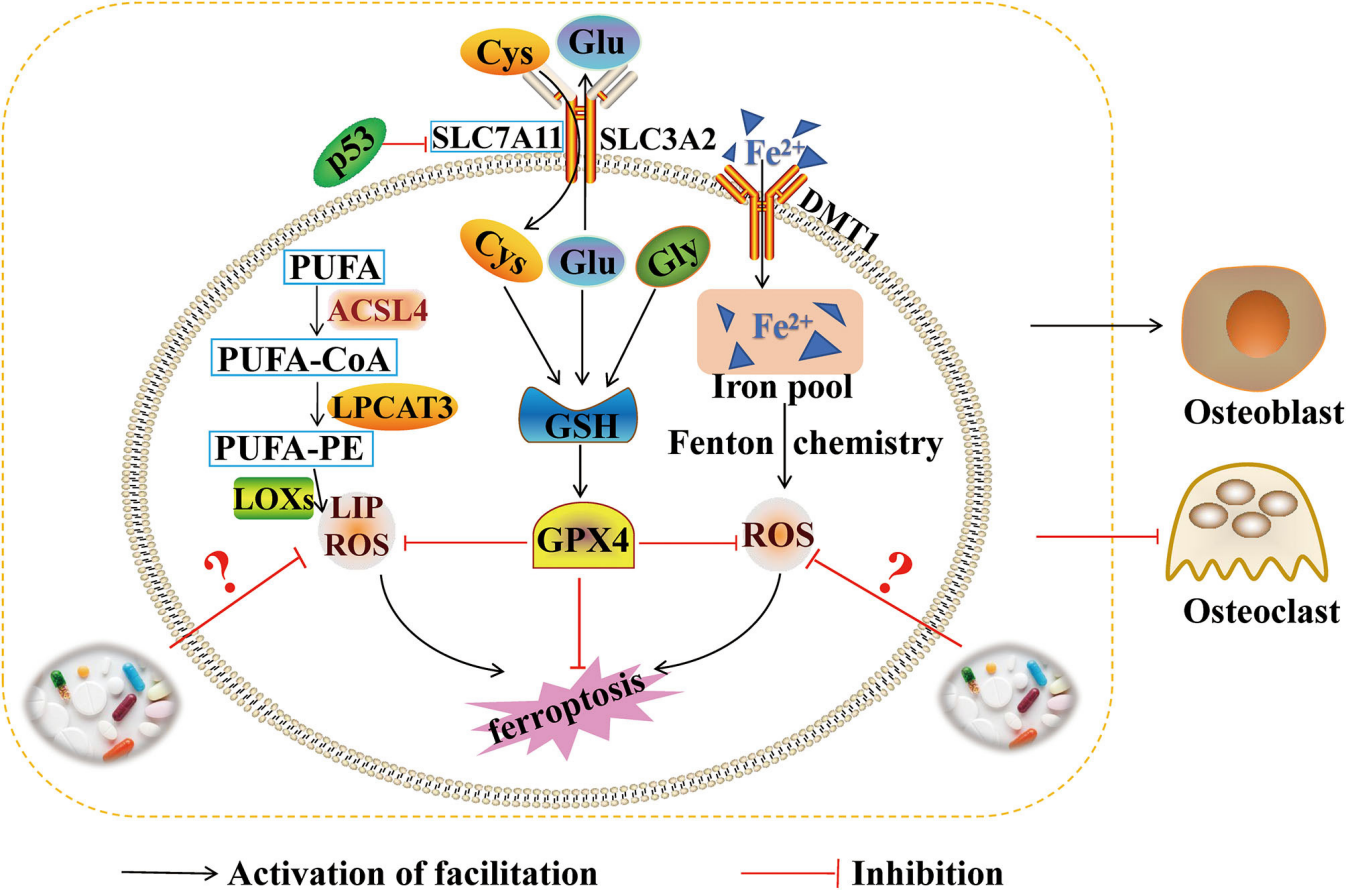
Ferroptosis and bone formation. This figure summarizes the molecular mechanism of ferroptosis and the regulatory effect of ferroptosis on bone formation. The black arrow represents the activation of the regulatory role of ferroptosis. The red inhibition arrow represents the inhibition of the regulatory role of ferroptosis and medicine.

Ferroptosis is an abnormal metabolic process, which is involved iron, lipids, and amino acids. Iron overload, GPX4/GSH, and lipid peroxidation are the main mechanisms leading to the steady state imbalance of iron overload. The pathogenesy of ferroptosis is very complicated. Each mechanism is independent of each other and related to each other, forming a complex network regulation. Recently, ferroptosis has become a research hotspot, and some progress has been made in osteoporosis research. During bone formation, abnormalities of iron transport-related proteins IRP2, FLT, FPN1, TFR1, TFR2, and HEP can lead to changes in iron concentration. Iron overload produces lots of ROS through the Fenton reaction, causing bone remodeling imbalance. In this mechanism, BMP/p38MAPK, BMP/SMAD, NRF2/HO-1, PI3K/AKT/FOXO3a/DUSP14, RIPK1/ RIPK3/MLKL, Wnt, and many other channels are participated in the regulation of ferroptosis and are accompanied by the inhibition of the antioxidant system GPX4/GSH. At the same time, ferroptosis may promote the expression of RANKL and enhance bone resorption, which was producing a large amount of ROS by Fenton reaction. Ferroptosis can also affect OC proliferation and differentiation, which was regulating the expression levels of NFATc1, c-fos and c-myc of MAPK signaling pathway. Therefore, RANKL may be a key target for regulating bone resorption during ferroptosis metabolism. However, the signal network mechanism regulated by RANKL has not been thoroughly studied. It will be the focuses of research for future studies on the etiology mechanism of osteoporosis. In this review, ferroptosis produces a large amount of ROS through Fenton reaction, and then a large amount of ROS leads to bone remodeling disorder by inhibiting the activity and differentiation of OB, and promoting the differentiation of OC, which fully proves that iron metabolism balance plays an important role in osteoporosis. However, this review does not provide a detailed summary of the specific molecular mechanisms by which ferroptosis affects bone remodeling in osteoporosis, the specific regulatory targets, whether there is crosstalk regulation between ferroptosis and osteoporosis, and what is the targets and mechanisms of crosstalk regulation between ferroptosis and osteoporosis. Further, the crosstalk regulatory targets and mechanisms of ferroptosis and osteoporosis have become an important research content and direction of osteoporosis (Figure 4).

## Author Contributions

CY, JZ, FA, JW, YS, LY, DL, YZ, and YW: writing and editing the manuscript. All authors contributed to the article and approved the submitted version.

## Funding

This present study was funded by the grants from the National Natural Science Foundation of China (82060872), Natural Science Program in Gansu Province (21JR11RA138 and 20JR5RA616), the Project of Improving the Employment and Entrepreneurship Ability of College Students in Gansu Province (6-1), and the Project of Health Science and Technology Development in Lanzhou City (2021004).

## Conflict of Interest

The authors declare that the research was conducted in the absence of any commercial or financial relationships that could be construed as a potential conflict of interest.

## Publisher's Note

All claims expressed in this article are solely those of the authors and do not necessarily represent those of their affiliated organizations, or those of the publisher, the editors and the reviewers. Any product that may be evaluated in this article, or claim that may be made by its manufacturer, is not guaranteed or endorsed by the publisher.
